# Impact of burnout and professional fulfillment on intent to leave among pediatric physicians: The findings of a quality improvement initiative

**DOI:** 10.1186/s12913-024-10842-2

**Published:** 2024-04-05

**Authors:** R. Thomas Collins, Aric Schadler, Hong Huang, Scottie B. Day, John A. Bauer

**Affiliations:** 1 Division of Cardiology, Department of Pediatrics, University of Kentucky College of Medicine, 138 Leader Ave, 40508 Lexington, KY USA; 2 Division of Neonatology, Department of Pediatrics, University of Kentucky College of Medicine, Lexington, KY USA; 3 Division of Critical Care Medicine, Department of Pediatrics, University of Kentucky College of Medicine, Lexington, KY USA

**Keywords:** Physician burnout, Professional fulfillment, Organizational support, Intent to leave

## Abstract

**Background and Objectives:**

Physician burnout is rampant, and physician retention is increasingly hard. It is unclear how burnout impacts intent to leave an organization. We sought to determine how physician burnout and professional fulfillment impact pediatric physicians’ intent to leave (ITL) an organization.

**Design and Methods:**

We performed 120, 1:1 semi-structured interviews of our pediatric faculty and used the themes therefrom to develop a Likert-scale based, 22-question battery of their current work experience. We created a faculty climate survey by combining those questions with a standardized instrument that assesses burnout and professional fulfillment. We surveyed pediatric and pediatric-affiliated (e.g. pediatric surgery, pediatric psychiatry, etc.) physicians between November 2 and December 9, 2022. We used standard statistical methods to analyze the data. An alpha-level of 0.05 was used to determine significance.

**Results:**

A total of 142 respondents completed the survey, 129 (91%) were Department of Pediatrics faculty. Burnout was present in 41% (58/142) of respondents, whereas 30% (42/142) were professionally fulfilled. There was an inverse relationship between professional fulfillment and ITL, *p* < 0.001 for the trend. Among those who were not professionally fulfilled, the odds ratio of ITL in the next three years was 3.826 [95% CI 1.575–9.291], *p* = 0.003. There was a direct relationship between burnout and ITL, *p* < 0.001 for the trend.

**Conclusions:**

Among pediatric physicians, professional fulfillment is strongly, inversely related with ITL in the next three years. Similarly, burnout is directly related with ITL. These data suggest a lack of professional fulfillment and high burnout are strong predictors of pediatric physician turnover.

**Supplementary Information:**

The online version contains supplementary material available at 10.1186/s12913-024-10842-2.

## Introduction

Recruitment and retention are ongoing challenges for many medical centers. In heterogeneous studies of physicians, intent-to-leave (ITL) in the next two years is reported by about a quarter of participants [1, 2], and most centers actually experience an annual physician attrition rate of 6-7.5% [3, 4]. Intent to leave has been shown to vary by subspecialty, with those in general pediatrics and pediatric subspecialties being among the lowest [5]. 

Intent to leave and actual departure from the organization are significantly increased among physicians experiencing burnout and decreased professional fulfillment [3, 5]. With physician burnout at epidemic proportions, affecting 40–50% of physicians in the United States [6], many centers are facing increased physician turnover. For those physicians later in their careers, attrition via early retirement has recently been driven by burnout, with some demonstrating a three-fold increase during the COVID-19 pandemic [7]. Compounding the issue of physician turnover and attrition is the association of burnout with decreased career engagement and professional fulfillment among physicians [8], which also are independently associated with increased ITL [9, 10]. 

While associations between physician burnout, professional fulfillment, and ITL have been investigated in some medical and surgical fields, little has been done to investigate them amongst pediatric physicians. In a large national study of various specialties, ITL was among the lowest levels for respondents in general pediatrics and pediatric subspecialties, though the relationship with burnout and satisfaction was not specifically analyzed among the pediatric physicians in the study [5]. Given this prior finding in conjunction with the limited data on the associations of burnout, professional fulfillment, and ITL among pediatric physicians, we sought to determine how burnout and professional fulfillment affect ITL among our pediatric physician faculty. These pediatric physician-specific data would be helpful in determining drivers for ITL and could be used to shape interventions to prevent potential loss of pediatric physicians from organizations and the medical workforce.

## Methods

The Institutional Review Board of The University of Kentucky reviewed the project and deemed it to be exempt from institutional oversight and granted a waiver for informed consent (IRB #86,919). All study methods were carried out in accordance with relevant guidelines and regulations.

From August 15, 2022 through October 20, 2022, one author (R.T.C.) conducted one-on-one interviews with 121 of the physician faculty in the Department of Pediatrics seeking to understand career goals and challenges the faculty were facing. Each faculty member was asked the same two questions: “What do you hope for your career in the next five to ten years?” and “What are the barriers you see to accomplishing that?” The author used inductive content analysis to identify themes from the interviews [11], and those themes were used to craft a 22-question battery assessing drivers of physicians’ perspectives (Supplemental material).

The 22-question battery was combined with the Stanford Professional Fulfillment Index, a validated instrument that assesses burnout, interpersonal disengagement, work exhaustion, and professional fulfillment, to create a faculty climate survey [12, 13]. All items on the Stanford Professional Fulfillment Index are scored on a 5-point Likert scale ranging from “not at all” to “extremely” for burnout-related items and “not at all true” to “completely true” for professional fulfillment items. The professional fulfillment scale “assesses the degree of intrinsic positive reward” derived from work, “including happiness, meaningfulness, contribution, self-worth, satisfaction, and feeling in control when dealing with difficult problems at work.” [12] The burnout scale assesses physical and emotional exhaustion; decreased empathy with colleagues and patients; and diminished connectedness with colleagues and patients [12]. Scores are normalized to a 0–10 scale with higher professional fulfillment scores being favorable and higher burnout scores being unfavorable [12, 14]. Overall burnout, work exhaustion, and interpersonal disengagement are defined as present when the score is ≥ 3.325, and professional fulfillment is defined as present when the score is ≥ 7.5 [12]. 

The faculty climate survey, composed of the 22-question battery and the Stanford Professional Fulfillment Index, was built and housed in a secure, online database (Qualtrics, Seattle, WA). All physician faculty at Kentucky Children’s Hospital were invited via a generic email notification to participate in the survey, and survey responses were anonymous. In addition to the data from the survey, participants provided data on academic rank, years of service at the University (i.e., < 3 years, 3–7 years, 8–12 years, > 12 years), primary clinical venue, gender, clinical division (optional), and comments they felt appropriate. The survey was open to responses from October 28, 2022 until December 9, 2022. To analyze ITL, we examined responses to the statement, “In the next three years, I think there is a high likelihood I will seek another career opportunity outside of the University of Kentucky.” We dichotomized ITL as a Yes/No by combining “somewhat agree” and “strongly agree” responses [15] and compared them to the rest (“neither agree nor disagree,” “somewhat disagree,” and “strongly disagree”) in order to simplify interpretation of the covariates with odds ratios using a multivariable logistic model.

Pearson chi-square and Fisher’s exact test were used to assess differences among frequencies for categorical study variables, as appropriate. Significant variables identified in the bivariate analysis were further analyzed using a multivariable logistic regression model utilizing a backward elimination variable selection methodology. An alpha level of 0.05 was used to identify significance in the model. IBM SPSS Statistics version 28 (IBM Corp., Armonk, NY) and SAS 9.4 (SAS Institute Inc, Cary, NC) were used for the bivariate analysis and multivariable modeling, respectively.

## Results

A total of 225 pediatric physicians were invited to participate in the survey, of whom 142 completed it (overall response rate of 63%). Of the 150 faculty in the Department of Pediatrics, 129 completed the survey (departmental response rate of 86%). There were no differences in any study measures (i.e., burnout, fulfillment, or ITL) between Department of Pediatric faculty respondents and other pediatric subspecialty respondents (e.g., pediatric surgery, orthopedics, radiology, psychiatry, etc.).

**Fig. 1:**
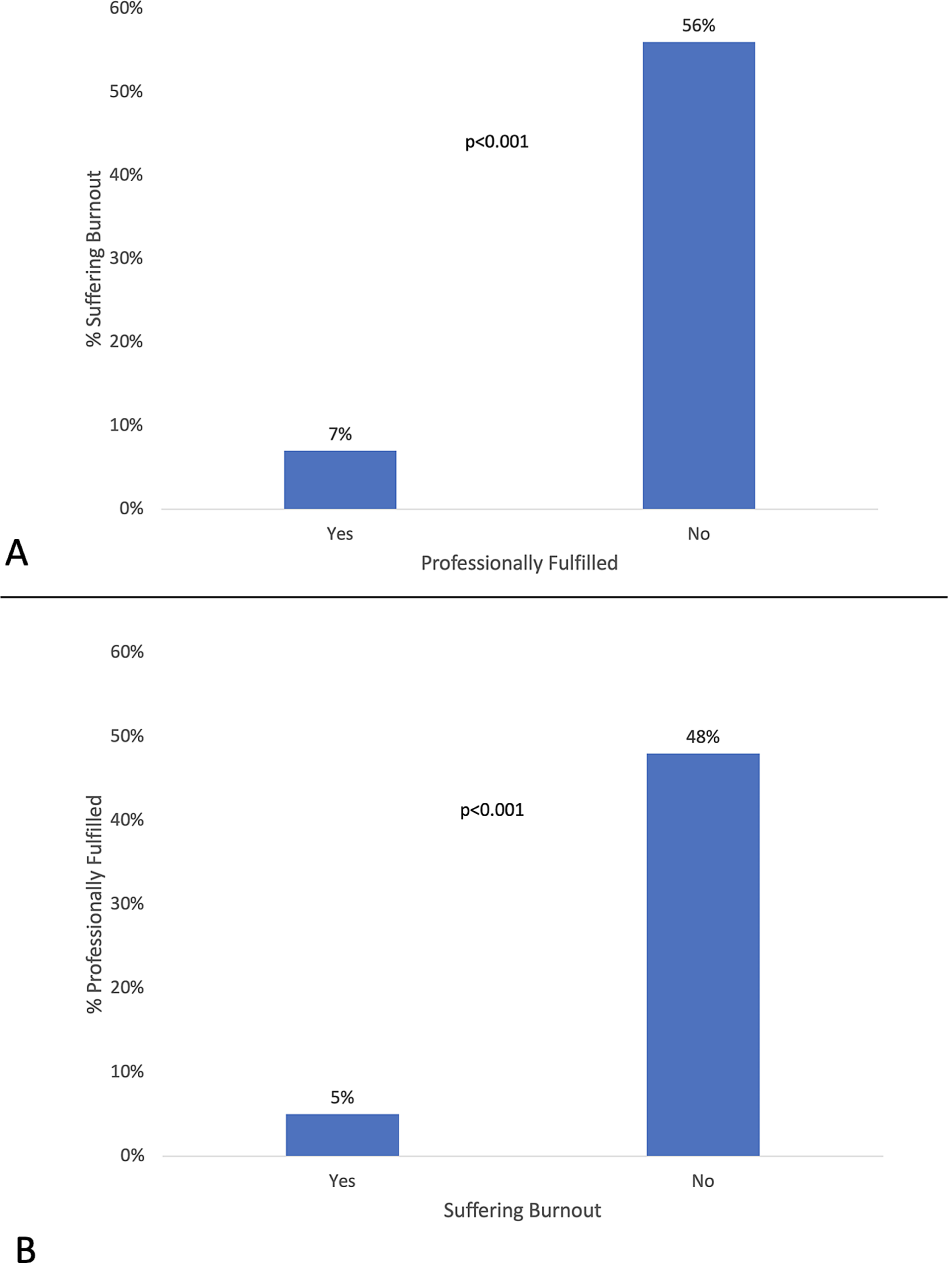
Relationships of Burnout and Professional Fulfillment. **Panel A** demonstrates that when pediatric physicians are professionally fulfilled, the prevalence of burnout is dramatically lower than when they are not fulfilled. Additionally, when fulfilled, burnout is substantially lower than nationally reported levels. **Panel B** demonstrates that when burnout is present, pediatric physicians are rarely professionally fulfilled, whereas fulfillment is nearly an order of magnitude more prevalent when they are not suffering burnout

Burnout was present in 62/142 (43.6%) of respondents. As shown in Table 1, burnout was associated with multiple study measures. The prevalence of burnout was significantly higher among mid-career faculty (i.e., Associate Professors) and those who had been at the institution for 3–12 years. Figure 1 demonstrates the relationships between burnout and professional fulfillment. Multivariable logistic regression confirmed this inverse association (OR: 0.099, 95% CI 0.041–0.24)(*p* < 0.0001). Burnout was directly related to perceived excessive workload (OR 3.02, 95% CI 1.27–7.18)(*p* = 0.0126) and feeling unappreciated (OR: 2.57, 95% CI 1.17–5.65)(*p* = 0.0189).

**Table 1:** Study Variables Associated with Pediatric Physician Professional Fulfillment and Burnout

Variable	Prevalence of Burnout	p-value	Prevalence of Professional Fulfillment	p-value
**Faculty rank**				
Assistant Professor (*n* = 62) Associate Professor (*n* = 44) Professor (*n* = 30)	24 (39%) 26 (59%) 5 (17%)	0.001	21 (34%) 13 (30%) 8 (26%)	0.68
**Years at the University of Kentucky**				
< 3 years (*n* = 34) 3–7 years (*n* = 38) 8–12 years (*n* = 24) >12 years (*n* = 42)	9 (29%) 24 (62%) 14 (58%) 11 (26%)	< 0.001	17 (50%) 4 (11%) 6 (25%) 15 (35%)	0.002
**Gender**				
Male (*n* = 67) Female (*n* = 56) Prefer not to say (*n* = 13)	21 (32%) 24 (43%) 11 (79%)	0.005	26 (39%) 15 (27%) 1 (8%)	0.058
**Feel appreciated**				
Yes (*n* = 83) No (*n* = 58)	26 (31%) 33 (57%)	0.002	34 (41%) 8 (14%)	< 0.001
**Perceived excessive workload**				
Yes (*n* = 95) No (*n* = 46)	53 (56%) 6 (13%)	< 0.001	18 (19%) 24 (52%)	< 0.001
**Perceived organization as supportive**				
Yes (*n* = 67) No (*n* = 74)	16 (24%) 43 (58%)	< 0.001	30 (45%) 12 (16%)	< 0.001

Professional fulfillment was present in 42/142 (29.5%) of respondents. Professional fulfillment was associated with multiple study measures (Table 1). The prevalence of professional fulfillment demonstrated an inverse pattern across years at the institution compared to burnout; fulfillment was markedly lower amongst those with 3–7 and 8–12 years compared to the other groups. Multivariable logistic regression confirmed this inverse association (OR: 0.12, 95% CI 0.049–0.295)(*p* = 0.0022). Professional fulfillment was directly associated with perceived organizational support (OR: 2.64, 95% CI 1.42–4.90)(*p* < 0.0001).

**Fig. 2:**
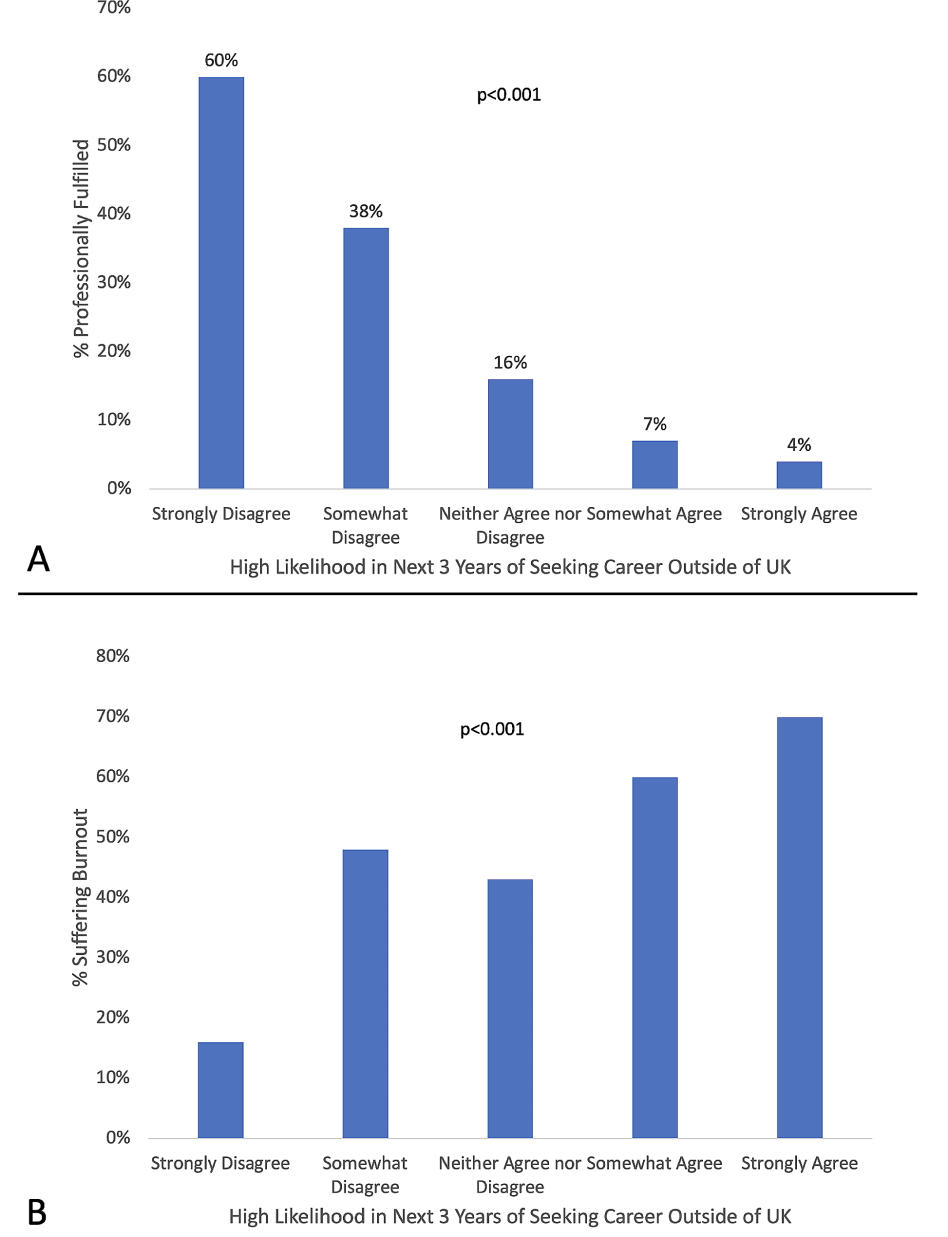
Relationship of Intent to Leave with Professional Fulfillment and Burnout. **Panel A** demonstrates that professional fulfillment is highly inversely correlated with intent to leave the organization within the next three years. **Panel B** demonstrates a direct relationship between intent to leave the organization and pediatric physician burnout

**Fig. 3:**
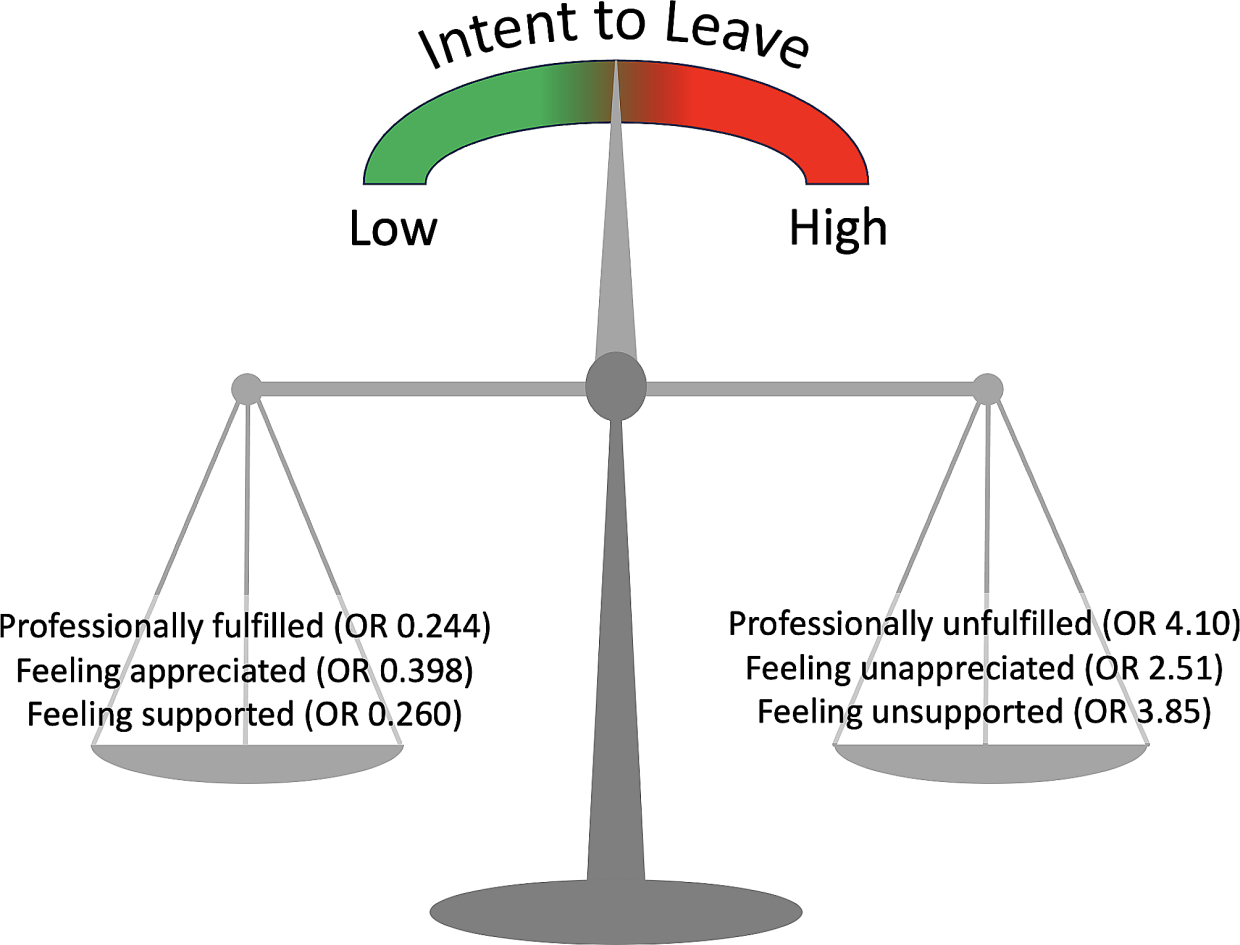
Interaction of Intent to Leave with Fulfillment, Appreciation, and Support. As professional fulfillment, feeling appreciated, and/or supported by the organization increase, the scale tips leftward so that intent to leave decreases. Conversely, as lack of professional fulfillment, feeling unappreciated, and/or unsupported by the organization increase, the scale tips rightward so that intent to leave increases. Numbers in parentheses represent the odds ratio for intent to leave relative to the variable in question

Intent to leave within the next three years was present in 38/139 (27.3%) of respondents. As shown in Fig. 2, ITL was directly related to burnout and inversely related to professional fulfillment. Multivariable logistic regression demonstrated inverse relationships among ITL and perceived organizational support (OR: 0.260, 95% CI 0.11–0.60)(*p* = 0.0017), professional fulfillment (OR: 0.244, 95% CI 0.082–0.72)(*p* = 0.011), and feeling appreciated (OR: 0.398, 95% CI 0.197-0.80)(*p* = 0.0102). Figure 3 displays a conceptual model of how these three factors interact with ITL. Compared to those with < 3 years at the institution, the OR for ITL was 0.27, 95% CI 0.10–0.71 (*p* = 0.0077) for those who had been at the institution for > 12 years. Otherwise, we did not identify an association between years of service and ITL.

## Discussion

In this study, we sought to analyze the association of physician burnout, professional fulfillment, and ITL among pediatric physicians in our academic medical center. There are three major findings in our study. First, physician burnout and professional fulfillment were highly related. Second, burnout and professional fulfillment were strongly associated with ITL within the next three years. Finally, organizational factors (i.e., perceived excessive workload, feeling unappreciated, and perceived organizational support) had significant impacts on physician burnout and professional fulfillment.

Our data demonstrate physician burnout and professional fulfillment are highly inversely correlated. This fits with the U.S. Surgeon General’s characterization of burnout as “an occupational syndrome characterized by a high degree of emotional exhaustion and depersonalization (i.e., cynicism), and a low sense of personal accomplishment at work.” [16] Professional fulfillment is rooted in factors such as “contribution” and “satisfaction,” which are closely tied to one’s sense of accomplishment. There is a close, inverse relationship between burnout and professional fulfillment in non-pediatric physicians [17, 18], and a study of pediatric physicians found a clear, inverse relationship between burnout and career satisfaction, which is closely related to career fulfillment [19]. Despite these patterns, our data indicate burnout and professional fulfillment are not antonymous. Professors in our cohort exhibited lower levels of both burnout and professional fulfillment, indicating the inverse relationship is not consistently maintained. It has been observed it is possible for both burnout and career satisfaction to be high, particularly when attributed to the professional rewards and inspiration that come from helping children [20]. However, this explanation is incomplete in elucidating the low level of career fulfillment amongst our Professors. This finding in our data requires further investigation.

Pediatric physician professional fulfillment and burnout were strongly associated with ITL the organization within the next three years. Multiple authors have found decreased job satisfaction [9, 10, 21] and professional fulfillment [17, 18] are associated with increased ITL and turnover amongst physicians in general. In Chinese pediatricians, job satisfaction was inversely related to ITL [22]. Our data in pediatric physicians affirms this relationship. Additionally, multiple prior authors studying non-pediatric physician cohorts have found that burnout is highly associated with ITL, with odds ratios from 2 to 5 for the relationship [5, 9, 10, 21, 23, 24]. Within the limited studies of pediatric physicians, among pediatric intensivists in our institution, severe burnout has been shown previously to be associated with considering leaving the organization, though that may represent a different mental outlook from those with ITL [25]. Our current data corroborate the relationship between physician burnout and ITL amongst pediatric physicians. Taken together, our data indicate, not only for our institution, but also for others, that increased levels of physician burnout and lack of professional fulfillment may drive increased physician turnover for affected institutions.

We found our participants’ perceptions of specific organizational factors, including perceived excessive workload, feeling unappreciated, and perceived organizational support, were associated with physician burnout and professional fulfillment. Various organizational factors contribute to burnout, encompassing issues related to electronic health records, inefficient work environments, poor organizational leadership, and culture [26]. Organizational-level interventions are more effective at curbing burnout than are individual-focused interventions, indicating organizational issues are the primary drivers of burnout [27]. Consistent with our data, increased workload heightened the risk of physician burnout [28]. Among adult critical care physicians, increased workload correlated with intensity of burnout, which, in turn, was associated with ITL [29]. The type of workload type has been shown to affect burnout and intent to leave differently. The type of workload also played a role, as both mental and physical workload among physiatrists were directly correlated with increased risk for burnout, but only physical workload was directly associated with ITL [30]. Beyond workload, feeling appreciated by one’s organization corresponds to increased professional fulfillment and decreased burnout. For instance, emergency physicians who felt appreciated displayed a 90% increased odds of professional fulfillment [18]. Among adult critical care physicians, burnout was lower among those who felt valued by their organization [31]. Feeling appreciated was similarly impactful amongst radiology trainees, reducing both burnout and ITL, with a 60% reduction in ITL associated with organizational appreciation [2]. Unlike organizational appreciation, fewer studies in physician groups have been published on the relationship of perceived organizational support with burnout and professional fulfillment. Recently, amid the COVID-19 pandemic, perceived organizational support was associated with decreased risk for burnout [32]. Physicians perceiving organizational support in implementing electronic health records reported lower burnout [33]. A supportive environment was also linked to decreased burnout and ITL among adult critical care physicians [31]. Additionally, work among nurses found that perceived organizational support was associated with lower risk of burnout [34] and ITL, as well as increased job satisfaction [35]. Little has been done to assess the relationship between organizational support and physician professional fulfillment. Among internal medicine physicians, satisfaction that their workplace is supportive has been associated with increased professional fulfillment [36]. Similarly, institutional support of academic interests has been positively correlated with physician job satisfaction [37]. Our data add to the literature by demonstrating clear associations of organizational support with pediatric physician context. Perception of organizational emerged as a strong predictor of ITL in our study, highlighting its importance for organizations aiming to retain physician faculty.

Because increased physician workload derives from multiple sources, creating a supportive environment requires assuring appropriate numbers of physicians, advanced practice providers, and ancillary and administrative staff, as well as decreasing the burden of documentation and the electronic medical record [38]. A supportive environment also involves assigning physicians proper responsibilities, implementing suitable work schedules, and allowing adequate time away from work [39]. Without these organizational elements, organizations are likely to have limited success in addressing ITL. Supportive environments foster feelings of being appreciated, and decreased workload opens time for other professionally fulfilling activities. When physicians have time to work on those things they find most fulfilling, their risk of burnout decreases [28]. Addressing these issues, which are associated with a lack of organizational support, will have direct positive effects on burnout and ITL. Consequently, we are actively implementing numerous mechanisms to improve organizational support—as well as physicians’ recognition of it—to address the issues identified in our work.

While our study has multiple strengths, it also carries some limitations. Ours was a single-center, cross-sectional study of pediatric physicians at a large, university-based medical center in the southeastern United States, which may have impacts on the applicability of our results. This limitation may be mitigated by the diversity of our faculty from across the U.S. and numerous other countries. Our study is subject to selection bias, as not all of those invited responded to the survey. However, the risk of this affecting our results is likely low given our relatively high departmental response rate and the lack of differences between those in the Department of Pediatrics and those outside of it. Our sample size is relatively small. Our data were anonymous, which limited our ability for further analyses, such as impact of age, ethnicity, or other demographic features. Multiple respondents preferred not to provide their gender or clinical division, which limits the specificity of some of our findings. We analyzed burnout and professional fulfillment as binary concepts, which may have impacts on our results.

## Conclusions

Among pediatric physicians, burnout and professional fulfillment are highly related, though not direct opposite states. Organizational support serves an instrumental role in mitigating physician burnout and increasing professional fulfillment. A lack of organizational support, coupled with perceived excessive workload appears to increase pediatric physician burnout and ITL that could have major negative impacts on healthcare organizations. We agree with numerous others that substantive organizational initiatives are central to arresting this process and changing the current physician burnout crisis.

### Electronic supplementary material

Below is the link to the electronic supplementary material.

Supplementary Material 1

## Data Availability

The deidentified study data are available upon request and approval from the authors.

## Abbreviations

ITL: Intent to leave
