# Supplementary material for: Impact of burnout and professional fulfillment on intent to leave among pediatric physicians: The findings of a quality improvement initiative
Source: BMC Health Serv Res. 2024 Apr 5;24:434. doi: 10.1186/s12913-024-10842-2 (PMC10998309; doi:10.1186/s12913-024-10842-2)
Supplement: Supplementary file 1 — Supplementary Material 1 [file 12913_2024_10842_MOESM1_ESM.docx]

Supplemental Tables

**Supplemental Table 1.** 22-Statement Battery Derived from Pediatricians’ Responses During Faculty Interviews

| Statement |
| --- |
| I feel appreciated for my contribution. |
| I do not feel like anyone considers my viewpoint. |
| I am highly fulfilled in my academic career here. |
| The clinical demands of my job negatively impact my teaching. |
| My clinical demands prevent me from doing research. |
| The expected level of clinical productivity on me is appropriate. |
| The RVU system is good for faculty. |
| Our clinical workload increases patient safety and quality. |
| Teaching and research are highly valued by the organization. |
| I chose academic medicine because teaching is important to me. |
| I chose academic medicine because I love conducting research. |
| The priorities of the organization reflect the tripartite academic mission. |
| I am concerned about the future of our academic program. |
| I do not feel my efforts to teach are recognized or appreciated. |
| My research work is noticed and appreciated by our organization. |
| We have excellent research resources available. |
| I feel like accessing research resources is really difficult. |
| Our research resources have made it easier for me to conduct research. |
| In the next 3 years, there’s a high likelihood I will seek to leave UK. |
| My likelihood of staying at UK has more to do with life outside of work. |
| I would recommend joining our faculty to a colleague who loves to teach. |
| I would recommend jointing our faculty to a colleague who loves to do research. |

**Supplemental Table 2.** Logistic Regression Models for Burnout, Fulfillment, and Intent to Leave

| Variable | Odds Ratio | 95% CI for Odds Ratio | p-value upon removal from model |
| --- | --- | --- | --- |
| Burnout Model | | | |
| Appreciation | 2.570 | (1.169, 5.653) |  |
| Workload | 3.016 | (1.267, 7.179) |  |
| Fulfillment | 0.099 | (0.041, 0.240) |  |
| Organization |  |  | 0.1891 |
| Years of service |  |  | 0.2452 |
| Gender |  |  | 0.3824 |
| Years of service*Gender |  |  | 0.4835 |
| Fulfillment Model | | | |
| Organization | 2.636 | (1.419, 4.897) |  |
| Burnout | 0.120 | (0.049, 0.295) |  |
| Appreciation |  |  | 0.1667 |
| Years of service |  |  | 0.3637 |
| Workload |  |  | 0.7753 |
| Gender |  |  | 0.9687 |
| Intent to Leave | | | |
| Fulfillment | 0.244 | (0.082, 0.723) |  |
| Years of service  (3-7 vs <3) | 0.841 | (0.205, 3.457) |  |
| Years of service  (8-12 vs <3) | 0.710 | (0.153, 3.289) |  |
| Years of service  (>12 vs <3) | 0.143 | (0.035, 0.583) |  |
| Appreciation | 0.398 | (0.197, 0.803) |  |
| Organization | 0.260 | (0.112, 0.603) |  |
| Burnout |  |  | 0.2714 |
| Gender |  |  | 0.3077 |
| Workload |  |  | 0.2711 |
